# New Insights in Recurrent HCV Infection after Liver Transplantation

**DOI:** 10.1155/2013/890517

**Published:** 2013-04-23

**Authors:** Shih-Hsien Hsu, Ming-Lun Yeh, Shen-Nien Wang

**Affiliations:** ^1^Graduate Institute of Medicine, College of Medicine, Kaohsiung Medical University, Kaohsiung, Taiwan; ^2^Hepatobiliary Division, Department of Internal Medicine, Kaohsiung Medical University Hospital, Kaohsiung, Taiwan; ^3^Division of Hepato-Biliary-Pancreatic Surgery, Department of Surgery, Kaohsiung Medical University Hospital, No. 100, Tzyou 1st Road, Kaohsiung 807, Taiwan; ^4^Department of Surgery, Faculty of Medicine, College of Medicine, Kaohsiung Medical University, Kaohsiung, Taiwan

## Abstract

Hepatitis C virus (HCV) is a small-enveloped RNA virus belonging to the Flaviviridae family. Since first identified in 1989, HCV has been estimated to infect 170 million people worldwide. Mostly chronic hepatitis C virus has a uniform natural history, from liver cirrhosis to the development of hepatocellular carcinoma. The current therapy for HCV infection consists of a combination of Pegylated interferon and ribavirin. On the other hand, HCV-related liver disease is also the leading indication for liver transplantation. However, posttransplant HCV re-infection of the graft has been reported to be universal. Furthermore, the graft after HCV re-infection often results in accelerated progression to liver failure. In addition, treatment of recurrent HCV infection after liver transplantation is often compromised by enhanced adverse effects and limited efficacy of interferon-based therapies. Taken together, poor outcome after HCV re-infection, regardless of grafts or recipients, poses a major issue for the hepatologists and transplant surgeons. The aim of this paper is to review several specific aspects regarding HCV re-infection after transplant: risk factors, current therapeutics for HCV in different stages of liver transplantation, cellular function of HCV proteins, and molecular mechanisms of HCV entry. Hopefully, this paper will inspire new strategies and novel inhibitors against recurrent HCV infection after liver transplantation and greatly improve its overall outcome.

## 1. Introduction

Hepatitis C virus (HCV) was a member of Flaviviridae family virus, and seven major genotypes (Genotype 1~7a) have been identified with distinct regional distribution patterns. HCV is a major cause of chronic hepatitis worldwide, and end-stage liver disease caused by HCV has increasingly become the leading indication for liver transplantation (LT). It has been well known that HCV reinfection following LT examined by HCV RNA detection using the polymerase chain reaction occurs almost universally [1]. The natural history of HCV reinfection is substantially changed after LT with accelerated rate of cirrhosis recurrence of 8–44% in 5–7 years [2]. It has been pointed out that HCV reinfects the liver graft at time of reperfusion intraoperatively [3]. The virus source is attributed to the blood itself with a high probability [4]. The viral load can return to the pretransplant values within 4 days after transplantation and may be influenced by the usage of corticosteroids [5]. Acute hepatitis occurs between 2–5 months after transplant, and it is characterized by acute lobular hepatitis [4]. In the early reinfection stage, the graft injury occurs only after 3 weeks. Chronic hepatitis is established about 6–12 months after transplantation. The stage of chronic hepatitis is characterized by a decrease of viral load and a pattern of immune-mediated injury. A variant form of posttransplant HCV infection is cholestatic hepatitis C that occurs in <10% of patients, frequently associated with high viral load and immunosuppression. Usually, it occurs within 1–6 months after transplant and can progress to hepatic failure in 3–6 months [6]. This form is characterized by very high viral load, cellular ballooning, low inflammation, and a Th2 intrahepatic immunological response. These features suggest that the liver lesion is due to a direct cytopathic injury caused by HCV.

To date, the absence of preventive strategy for HCV reinfection after transplant is a major challenge for the HCV recipients undergoing LT. As mentioned above, reinfection of the liver graft is universal and characterized by accelerated progression of liver disease. Furthermore, treatment of recurrent HCV infection after LT is compromised by enhanced adverse effects and limited efficacy of interferon-based therapies. In addition, poor outcome after graft reinfection of HCV has increasingly become a major problem faced by the hepatologists and transplant surgeons. Thus, novel preventive and therapeutic strategies of HCV reinfection are urgently needed.

## 2. Risk Factors for HCV Recurrence following Liver Transplantation (LT)

Recurrence of HCV infection in the liver allograft is universal after LT, and its natural history is variable. It has been estimated that approximately 20% of recipients will progress to graft cirrhosis within 5 years of transplant [7]. Overall, HCV disease is more aggressive in the posttransplant recipients than in patients whose immunity is intact [8]. Accelerated disease progression is multifactorial and probably depends on a number of variables, including host, donor, viral, and external factors. However, the definite interactions between these factors and recurrent HCV infection in the liver allograft still remain controversial and poorly defined. Thus, to identify recipients at risk for rapid HCV recurrence after LT will be helpful especially when considering treatment with the currently available antiviral agents either as prophylaxis or therapy. To date, a number of risk factors have been mentioned regarding this clinical issue.

### 2.1. Nonviral Factors

One study, reviewing 307 patients who underwent LT for HCV over a 10-year period, suggested that advanced donor age, prolonged donor hospitalization, increasing recipient age, and elevated recipient MELD scores were found to increase the relative risk of HCV recurrence [9]. Moreover, earlier studies have advocated that HCV recurrence may be more severe when older donors are used [10, 11]. In addition, the type of donor used may have an impact on HCV reinfection of the graft after LT. One clinical observation suggested that HCV recurrence is more severe in living donor LT compared to cadaveric LT [12]. However, another study reported that there are no differences observed in hepatitis C recurrence rate, severity of intrahepatic pathology, or graft and patient survival between living donor LT and cadaveric LT recipients [13]. As to the source of HCV reinfection in the allograft, it may occur at time of reperfusion intraoperatively [3]. The blood itself has a high probability to be the origin of this allograft reinfection [4]. It is indeed valuable that identifying these risk factors before LT, particularly when matching donors to HCV recipients, may decrease the incidence of HCV recurrence after LT.

### 2.2. Viral Load

HCV infection of the allograft is believed to be an extremely dynamic process. HCV virus binds to the new allograft at the time of reperfusion, and viral replication occurs within hours after LT. The impact of recipients' viral load on HCV recurrence following LT is still uncertain, but a clear understanding of HCV kinetics after LT will contribute to the development of strategies to prevent HCV infection of the allograft. An earlier study, which analyzed HCV kinetics during and immediately after LT, found a sharp decrease in HCV viral load during the anhepatic phase and immediately after graft reperfusion [3]. This decrease was presumably attributed to massive binding of HCV to the hepatocytes. In other words, reinfection is immediate after reperfusion. Powers et al. also reported that HCV RNA levels dropped with an average half-life of 0.8 hours during the anhepatic phase [14]. The authors also mentioned that viral loads then continued to drop up to 23 hours after implantation, and began to rise as soon as 15 hours after the anhepatic phase. Moreover, the viral load reached a plateau before rising, suggesting that a nonhepatic source supplied virions and balanced their intrinsic clearance. It was estimated that nonhepatic sources were at most responsible for 4% of total viral production and the remaining 96% occurred in the liver. Other similar studies pointed out that viral loads in serum returned to pretransplant levels within 24 to 48 hours after the procedure. Within weeks after transplant, viral loads would be approximately 1 log higher than pretransplant levels [4, 15].

However, studies investigating the impact of pretransplant viral load on the likelihood of HCV recurrence have produced controversial results. Several reports indicated that pretransplant viral load did not correlate with either the likelihood or timing of HCV recurrence following LT [16]. By contrast, one study including 166 HCV-infected recipients demonstrated that before transplant, HCV viral load greater than 1 million viral equivalents/mL was associated with decreased graft and patient survival [17]. Given the hypothesis that pretransplant treatment of HCV will reduce or prevent HCV posttransplant recurrence, it has been advocated that patients with HCV infection on the transplant list should be considered for therapy with the goal of obtaining sustained virological response (SVR) prior to LT [18].

### 2.3. HCV Genotype

The influence of HCV genotypes on HCV recurrence after LT had been assessed, but the results were still controversial. Some studies demonstrated that the severity of recurrence and levels of viral replication for HCV hepatitis after LT were higher in patients with genotype 1b HCV infection than other genotypes [7, 19–21]. Another study by Gayowski et al. reported in their results that the rate of recurrent HCV hepatitis, timing to recurrence, severity of recurrence and response to IFN therapy did not differ among genotypes and suggested that HCV genotype may not be a significant factor influencing post-LT HCV hepatitis [22].

### 2.4. *IL28B* Polymorphism

Recently, genetic variation in the region of the *IL28B* gene on chromosome 19, coding for IFN-*λ*3, has been demonstrated to be strongly associated with SVR in patients with genotype 1 chronic HCV infection who are treated with pEG-IFN plus RBV in the nontransplant setting [23, 24]. *IL28B *polymorphism has also been associated with spontaneous HCV clearance [25]. One cohort study, which included 189 consecutive HCV patients undergoing LT, aimed to determine the prevalence and impact on clinical outcomes of donor and recipient *IL28B* genotypes among liver transplant recipients [26]. The authors suggested that recipient *IL28B* TT genotype is associated with more severe histological recurrence of HCV after LT and CC donor grafts might be preferentially allocated to recipients with HCV infection.

### 2.5. CMV and HHV-6 Infection

Reactivation of herpes group viruses may also be one of the factors associated with HCV recurrence after LT. Cytomegalovirus (CMV) and human herpes virus-6 (HHV-6) are herpes viruses that commonly reactivate after transplantation. They are proposed to have an immunomodulatory effect in transplant recipients and may play a role in promoting HCV replication [27, 28]. Recently, a retrospective study of 347 first LT recipients (donor or recipient CMV seropositive) transplanted for HCV was performed to evaluate the associations of CMV infection and disease with recurrent hepatitis C after LT. It was found that CMV infection was associated with increased risk of fibrosis stage ≧2 and inflammation grade ≧2 [29]. However, their effects on posttransplant HCV recurrence still remain questionable. Data showed that short-term CMV viremia does not enhance the replication of HCV after LT [30].

### 2.6. Immunosuppression

Early data, examining the pathogenesis of graft injury in liver transplant recipients with HCV infection, first reported that methylprednisolone treatment for acute rejection led to a 4-100-fold increase in serum HCV RNA [31]. Since then, an aggravated course of HCV reinfection after LT and increased resistance to antiviral therapy have been attributed to the application of specific immunosuppressive medication [32, 33]. Herein, various immunosuppression strategies have been evaluated for their influence on HCV recurrence.

*Glucocorticoids. *Glucocorticoids are often given as an induction protocol during LT, and low doses combined with other immunosuppressants are used as maintenance immunosuppression after operation. In case of acute rejection, recipients receive pulse methylprednisolone to reverse the rejection. There are convincing data that bolus doses of glucocorticoids given for rejection treatment have a negative impact on HCV recurrence. It is estimated that cumulative exposure to corticosteroids is associated with increased mortality, higher levels of HCV viremia, and more severe histological recurrence [34]. One recent study demonstrated that glucocorticosteroids specifically increased HCV entry by upregulating the cell entry factors occludin and scavenger receptor class B type I. The data suggested that the potential effects of high-dose glucocorticosteroids on HCV infection *in vivo* may be due to increased HCV dissemination [35]. Previous studies have indicated that the specific CD4 T cell response to HCV is important in viral clearance of acute HCV infection after liver transplantation. Moreover, plasmacytoid dendritic cells are capable of producing large amounts of IFN*α* against HCV infection in this specific CD4 T cell response [36, 37]. Experimental data have shown that prednisolone suppressed the functions of plasmacytoid dendritic cells by promoting their apoptosis [38]. Thus, in a transplant setting, the consensus is that steroid avoidance or slow tapering of the dose is associated with reduced HCV recurrence [39, 40], whereas boluses for treating acute rejection can increase the viral load [31].

*Calcineurin Inhibitors (CNIs). *Cyclosporine is a lipophilic cyclic peptide of 11 amino acids, while tacrolimus is a macrolide antibiotic. Both drugs bind with high affinity to a family of cytoplasmic proteins (also called immunophilin), which present in a variety of immune cells. Immunophilin-dependent signal transduction via calcineurin represents a key event in the activation of T cell proliferation by regulating expression of the gene that encodes IL2. Cyclosporine A (CyA) binds to cyclophilin, while tacrolimus (Tac) binds to FK binding proteins (FKBPs). The binding blocks the phosphatase activity of calcineurin and subsequently inhibits TCR/CD3-induced T cell proliferation by the blockage of IL2 production. Intriguingly, in addition to their promoting role in calcineurin signalling, immunophilins are catalysts of protein folding and contribute to the invasion ability of several coronaviruses [41]. As to HCV, it is well established that cyclophilins have an important role in viral replication and *de novo* virus production. Recent data suggest that HCV replication is dependent on the interaction between cyclophilin B and nonstructural protein 5B (NS5B, HCV RNA polymerase) to stimulate its RNA binding activity and thereby promote the *de novo *synthesis of positive and negative stranded RNA [42]. Given the fact that immunophilins can work as a supportive role in viral infection, it is rational to find out that CyA is capable of the anti-HCV activity through mediating a specific blockade of cyclophilins to NS5B RNA [43]. As CyA interacts with both cyclophilin A and cyclophilin B, it is conceivable that CyA affects multiple steps in the life cycle of HCV. However, there is controversy about the anti-HCV effects of cyclosporine A *in vivo*. By contrast, Tac does not have any anti-HCV activity [44].

*MMF and MPA. *Mycophenolate mofetil (MMF) belongs to the class of antimetabolite immunosuppressive agents. Mycophenolic acid (MPA), the active metabolite of MMF, is a selective noncompetitive inhibitor of inosine monophosphate dehydrogenase (IMPDH). In addition to its potent immunosuppressive capacity, the *in vitro* assay has indicated that MPA has antiviral effect against HCV [45]. In HCV cell culture models, MPA could induce the expression of important antiviral interferon-stimulated genes, including interferon regulatory factor (IRF) 1, IRF 9, and interferon-induced transmembrane protein 3 (IFITM3) [46]. Among these proteins, data showed that IRF1 was directly involved in the anti-HCV activity of MPA. Moreover, MPA could have effects in synergy with IFN-*α* on HCV replication in the same HCV experiments. In addition, when combined with IFN-*α*, MPA augmented the transcription of multiple interferon-stimulated genes. With the molecular basis of how MPA works in synergy with IFN*α*, proper prospective clinical studies are warranted to confirm their synergistic effects against HCV *in vivo*. On the other hand, although the safety and efficacy of MMF as an immunosuppressive medication in HCV patients undergoing LT have been demonstrated, the exact effects on HCV recurrence have not been clearly studied.

*Rapamycin.* With low nephrotoxicity and potential anticancer properties, rapamycin has been increasingly used in the transplantation context [47]. It has been well known that rapamycin engages the cytosolic protein FKBP12 to form a complex. This complex inhibits the mTOR pathway by directly binding to the mTOR complex 1, resulting in blockage of cell cycle progression from the G1 to S phase and thereby causing inhibition of T cell proliferation. Intriguingly, rapamycin induces autophagy through inhibiting mTOR. Autophagy is a process for catabolizing organelles and other intracellular components to balance cellular metabolism and to promote cell survival during stressful conditions. In fact, autophagy is also an important event in the regulation of the cellular response against viral infections [48]. It is noteworthy that HCV infection induces autophagy in the hepatocytes via the unfolded protein response, and the autophagy induced by HCV is incomplete through blocking the maturation of autophagosomes to autolysosomes [49]. The autophagosomes will not be degraded but instead support viral replication. Recent study demonstrated that NS5B could directly interact with the host proteins to induce the autophagy [50]. In addition, the HCV-induced autophagy may promote infection by reducing the innate immunity [51]. Based on these findings, it is conceivable that rapamycin could affect HCV recurrence and antiviral interferon therapy. However, clinical evidence is needed to make sure of its *in vivo* effect.

## 3. Updated Strategy to Recurrent HCV Infection after LT

HCV reinfection of the graft is almost universal among recipients with active infections at the time of transplantation [52]. An accelerated progression of fibrosis is noted in those recipients with recurrent HCV after transplant, and at least 25 to 30% of patients will eventually develop liver cirrhosis within 5 to 10 years [53, 54]. Once liver cirrhosis is established, the first episode of decompensation may occur in as high as 40% of patients within 1 year. Apart from the rapid progression of fibrosis, recipients with recurrent hepatitis C may also develop severe fibrosing cholestatic hepatitis, characterized by jaundice, rapidly after organ transplantation in the absence of biliary obstruction causing a very high risk of graft failure [55]. For the reason, HCV-related recipients show a worse posttransplant outcome compared to HCV-negative recipients. A previous study which retrospectively analyzed 11036 patients with 11791 liver transplants confirmed that the HCV infection significantly impaired patient and graft survival after LT [56]. Focusing on the strategy to recurrent HCV infection after LT, three approaches have been identified according to the timing of treatment: pretransplantation antiviral therapy, posttransplantation prevention and preemptive treatment, and treatment for established reinfection.

### 3.1. Pretransplantation Antiviral Therapy

Pretransplant treatment to suppress HCV viraemia in patients on the waiting list may reduce the risk of graft reinfection. However, the aim of pretransplant antiviral therapy should be to achieve negative serum HCV RNA at transplantation, not to reduce the viral load only. Currently, Pegylated interferon (IFN) plus ribavirin (RBV) combination therapy is the standard of care for chronic hepatitis C. However, the treatment has shown to be limited in patients with decompensated cirrhosis because of the frequent and severe treatment-related complication, and only a few studies investigated the role of the current SOC in this patient group. Recently, Carrion et al. reported the results of Pegylated IFN plus RBV therapy in 51 patients awaiting LT in comparison to 51 untreated controls [57]. Most of the patients were HCV genotype 1, and the median duration of therapy was 15 weeks until LT. Over 80% of patients were Child-Pugh A/B. As a result, 29% of patients achieved negative-HCV RNA at transplantation, and 20% had an SVR after LT. Another study using conventional IFN plus RBV for a longer duration (6–12 months) showed a similar SVR rate of 26% after LT [58]. It is noteworthy that none of the patients who achieved SVR before LT developed graft reinfection in the study. In addition to the relative poor efficacy, safety of pegylated IFN is another concern in patients with decompensated cirrhosis. Carrion et al. reported that 43%, 29%, and 8% of patients suffered from discontinuation, decompensation, and death during therapy [57]. Although the incidence of decompensation and death is similar in comparison to control group, treated patients suffered from a significantly higher rate of bacterial infection than control group. Child-Pugh B/C is the only independent factor associated with bacterial infection. These data suggested that patients with decompensated cirrhosis should be closely monitored during Pegylated IFN therapy and followed by experts with considerable experience. Direct acting antivirals (DAAs), including protease, polymerase or other nonstructural protein inhibitors, are the newly developed agents for HCV treatment. Current data on HCV treatment using DAA are limited in patients with cirrhosis. In the phase III trials with telaprevir and boceprevir, there were only a few patients with advanced fibrosis or compensated cirrhosis included [59–61]. Although the results from the phase III trials of telaprevir and boceprevir triple therapy showed a higher SVR rates compared to Pegylated IFN/RBV therapy in advanced fibrosis and cirrhotic patients, the severe advanced events rates were also increased in patients receiving triple therapy. The data from compassionate use of protease inhibitors in viral C cirrhosis (CUPIC) cohort also demonstrated an increased serious adverse events rates (from 30% to 51%) and discontinuation rates (from 7% to 12%) in cirrhotic nonresponders compared to the phase III trials. Another study in 20 cirrhotic patients awaiting LT demonstrated that 71% of patients achieved undetectable HCV RNA at 12 weeks with DAA triple therapy. However, 25% of patients suffered from early discontinuation, and 10% of patients had decompensation during therapy. The results suggest that triple therapy must be administered cautiously with intensive safety monitoring in cirrhotic patients.

### 3.2. Posttransplantation Prevention and Preemptive Treatment

Because of the accelerated clinical course of recurrent hepatitis C after LT, strategy to prevent reinfection of the graft is needed. Antihepatitis B immunoglobulin had been approved to successfully prevent HBV recurrence after LT. It is used in antibody therapy for HCV-infected recipients. However, recent studies using hepatitis C immunoglobulin or monoclonal antibody show only transient decrease of liver HCV RNA levels in liver transplant recipients [62, 63]. Preemptive or early posttransplant antiviral therapy aims to prevent the rapid development of chronic hepatitis before there is evidence of recurrent HCV infection. It is usually initiated within one month after LT. Compared to immunocompetent subjects, antiviral therapy response decreases during this period because of the high level of immunosuppression. A pilot study by Mazzaferro collected 36 HCV-RNA^+^ cirrhotic patients who started a 12-month IFN plus RBV combination therapy within 3 weeks after LT [64]. The sustained virological response (SVR) was achieved in 33% of patients. None of the patients developed graft rejection, and normal histology was also noted in patients with SVR after a median followup of 52 months. However, higher than 26% of graft rejection was noted in another study using IFN and RBV combination therapy for more than 12 months [65]. Moreover, four other studies using different regiments, two IFN monotherapy and two pegylated—IFN monotherapy, showed a poor effect of less than 20% of SVR and a higher rate of treatment discontinuation and graft rejection [66–69]. Therefore, more prospective and large-scaled studies are still needed to investigate the most appropriate regimen for Preemptive treatment.

### 3.3. Treatment for Established Reinfection

Owing to relatively poor efficacy and high adverse effect, most experts now delay antiviral therapy till a histological evidence of recurrent HCV infection is established after LT. However, for patients diagnosed with fibrosing cholestatic hepatitis, antiviral therapy must be given as early as possible after LT because of the poor short-term prognosis and rapid fibrosis progression. Initially, conventional IFN-based therapy was used to treat recurrent HCV infection after LT. A systematic review, consisted of 27 studies with a total of 689 patients, demonstrated a mean SVR rate of 24% with conventional IFN-based therapy [70]. Compared to conventional IFN-based therapy, pegylated IFN-based therapy showed a significant improvement of SVR rate. Three recent systematic reviews demonstrated 20–40% of SVR rate in genotype 1 and 50–100% in genotype 2/3 subjects using Pegylated IFN-based therapy for recurrent hepatitis C after LT [71–73]. The same result is also found in the histological improvement, regardless of necroinflammation or fibrosis. The factors to predict a better SVR are also the same as in nontransplant patients. Genotype non-1, low pretreatment HCV RNA, rapid virological response, and adherence to therapy have been identified as the positive predictors [70–72]. The association between response and recipient/donor liver *IL-28B* genotype is also reported recently [26, 74]. Charlton et al. reported that recipient and donor liver *IL-28B* polymorphism were independent predictors of SVR. Patients with donor and recipient liver *IL-28B* CC genotype have highest SVR rates, whereas patients with donor and recipient liver *IL-28B* non-CC genotype have minimal SVR rates [26]. Fibrosing cholestatic hepatitis is another poor predictor, and it is almost incurable with IFN treatment when fibrosing cholestatic hepatitis develops [75]. Safety and tolerance for therapy are also among the major concern in LT recipients with HCV recurrence. Dose reduction, discontinuation, and acute rejection were noted in 44% (38–50%), 24% (21–27%), and 2% (1–3%) of patients treated with conventional IFN-based therapy [70]. For the patients treated with Pegylated IFN therapy, a better SVR rate was noted in comparison to the IFN-based group. Meanwhile, the rejection rate was also found to be elevated with a mean rate of 5–10% (highest 25%). In addition, dose reduction was noted in over 50% of patients, and treatment discontinuation was found in around 30% of patients [70–72].

In treatment of genotype 1 HCV infection, the first generation of DAAs, telaprevir and boceprevir were recently used to enhance the efficacy of the standard regiment, Pegylated IFN plus RBV [76]. Although DAAs significantly increase the SVR rate in naïve patients, relapsers, and nonresponders [59–61], evidence is still limited about their efficacy in recurrent HCV infection after LT. In addition, there are some limitations for safety and tolerance. One critical limitation is the potential interaction with CNI. One recent study in healthy volunteers showed that boceprevir increased the area under the curve of CsA and Tac by 2.7 and of 17, respectively, [77]. Another study reported that the concomitant administration of immunosuppressive therapy with telaprevir in healthy volunteers increased CsA and Tac exposures by approximately 4.6-fold and 70-fold, respectively [78]. In 2012, several teams have communicated preliminary results of DAAs use after LT, which are summarized by Coilly et al. [79]. Overall, at 12 weeks of triple therapy, a complete early virological response (EVR) was obtained in 71% of patients treated with boceprevir and in 73% of patients treated with telaprevir. Notably, the CsA dose should be reduced by 1.3-fold with boceprevir and fourfold with telaprevir. The Tac dose should be reduced by fivefold with boceprevir and 35-fold with telaprevir. It was concluded that DAAs could completely change the prognosis of patients with severe HCV recurrence and offer new perspectives for the treatment of HCV recurrence in liver transplant patients.

## 4. Current Knowledge on HCV Entry into Hepatocytes

The current therapy against HCV infection, consisting of a combination of pegylated interferon (IFN) and ribavirin (RBV), is limited by resistance, adverse effects, and high costs. The absence of preventive regiment is a major limitation for patients undergoing LT for HCV-related liver failure. Moreover, accelerated progression to liver cirrhosis after graft reinfection and limited efficacy of IFN-based therapies for recurrent HCV are two major clinical issues leading to poor posttransplant outcome of the HCV recipients [80]. Thus, novel preventive and therapeutic antivirals are urgently needed. Viral entry is required for initiation, spread, and maintenance of infection. HCV entry is a multistep process orchestrated by a number of viral and host cell factors. These factors include the viral envelope glycoproteins E1 and E2, CD81, scavenger receptor B1, and tight junction proteins claudin-1 and occluding. A clear understanding of the detailed mechanisms of HCV entry may shed light in the ways to prevent the liver graft from HCV recurrence.

### 4.1. HCV Evades Host Immune Responses and Enters Target Cells through E2

Humoral responses are often thought to play a key role in controlling HCV infection [81]. In clinical setting, patients spontaneously resolving from acute HCV infection tend to have an early induction of neutralizing antibody response. In contrast, chronic infection patients have a delayed initiation of neutralizing antibody response. Moreover, the variety of strategies the virus evolves to escape antibodies-mediated neutralization, including the accelerated evolution and the diversity of HCV [82]. Overall, the capacity of HCV to mutate continuously allows a high plasticity and an ability of the virus to adapt to variable environmental conditions and escape the host's immune responses leading to HCV persistene.

HCV entry to the hepatocytes requires interaction of its viral envelope glycoproteins (E1 and E2) and the cell membrane [83]. Both E1 and E2 contain putative fusion domains [84]. While little is known about the role of E1 in HCV entry, several domains in E2 have been defined to bind to CD 81 and scavenger receptor class B type I (SR-BI), two important host factors (described in the next paragraph). These interactions through E2 glycoprotein help HCV evade host immune responses and enter the hepatocytes. For example, hypervariate region 1 (HVR1), the first 27 amino acids of E2, plays an important role in viral fitness. It has been demonstrated that HVR1 was involved in SR-BI-mediated HCV entry [85]. Therefore, HVR1 has been suggested to be a target for neutralizing antibodies, but its high variability results in poor cross-neutralization potency of antibodies across different HCV isolates [86]. It is well known that host humoral responses are thought to play a critical role in controlling HCV infection. Several studies reported that broadly neutralizing antibodies might be directed against conserved conformational epitopes within E2 and mostly inhibit E2-CD81 interaction [87, 88]. Currently, new conformational and conserved epitopes have been identified in the N-terminal part of E2 [89]. The conserved nature of these epitopes holds great promise for the development of potent inhibitors for HCV entry.

### 4.2. Host Factors Adopted by HCV Entry

Several cell surface molecules of the target cells have been identified to interact with HCV, including CD81, the LDL receptor, highly sulfated heparan sulfate (HS), SR-BI, and claudin-1 (CLDN1), occludin (OCLN).

Cd81, a ubiquitously expressed 25 kDa tetraspanin, has been demonstrated to interact with the soluble truncated form of HCV E2 [89]. Its large extracellular loop (LEL) seems to play a critical role in this interaction process. Moreover, studies have identified several amino acid residues throughout the CD81 LEL and HCV E2, which might be crucial for E2-CD81 binding [90, 91]. SR-BI, an 82 kDa glycoprotein with a large extracellular loop, is highly expressed in the liver [92]. Physiologically, SR-BI binds a variety of lipoproteins (e.g., HDL and LDL) and is involved in bidirectional cholesterol transport at the cell membrane. The LEL of SR-BI has been demonstrated to interact with E2 HVR1 [93]. Recent studies suggest that the interplay between lipoproteins, SR-BI, and HCV envelope glycoproteins is required for HCV entry [94, 95]. CLDNs are critical components of tight junctions, which regulate paracellular permeability and polarity. CLDN1, a 23 kDa four-transmembrane protein, is predominantly expressed in the liver [96]. Intriguingly, CLDN1 may localize to the tight junction and the basolateral surfaces of hepatocytes [97]. Recent studies suggest that nonjunctional CLDN1 may be also involved in HCV entry [48, 98]. OCLN is a 65 kDa four transmembrane protein expressed in the tight junction of polarized cells. Several studies have shown that OCLN is probably involved in the late postbinding event of HCV entry [99, 100]. As HCV circulates in the blood in association with LDL and VLDL, the LDL receptor has also been proposed as an attachment and/or entry factor for HCV [100]. The LDLR has been shown to mediate the internalization of serum-derived HCV into CD81-deficient HepG2 cells by binding virus-LDL particles [101]. Since cholesterol has been shown to be necessary for HCV cell entry, it is rational that other cholesterol-uptake receptors might also have a role in this process. More recently, Niemann-Pick C1-like 1 cholesterol absorption receptor (NPC1L1), a 13-transmembrane-domain cell surface cholesterol- sensing receptor, has been demonstrated as a new HCV entry factor [102].

Using a functional RNAi kinase screen, recent study identified epidermal growth factor receptor (EGFR) and ephrin receptor A2 (EPHA2), two receptor tyrosine kinases (RTKs) which regulate a number of key cellular functions, as host factors for HCV entry [103]. The results showed that the identified RTKs mediated HCV entry by regulating CD81—claudin-1 coreceptor associations and viral glycoprotein-dependent membrane fusion, implying that inhibition of RTK function may constitute a new approach for the prevention and treatment of HCV infection.

### 4.3. Molecular Mechanisms of HCV Entry

HCV attachment and entry into the host cells is a complex and multistep process. This process is tightly orchestrated by several viral and host factors. In brief, HCV is believed to first interact with HS and LDLR on the membrane surface of hepatocytes to allow concentration of the virion. Subsequently, interaction with other host factors such as SR-BI, CD81, CLDN1, and OCLN ultimately leads to viral internalization via the so-called clathrin-mediated endocytosis. Fusion between viral and endosomal membranes is followed by release of the viral genome into the cytosol, where translation and replication take place. Finally, HCV particles are assembled and released from the hepatocytes.

Recently, accumulated data have tried to unravel the potential molecular mechanisms of HCV entry. Some fluorescence resonance energy transfer (FRET) studies described the critical role of the association between CD81 and CLDN1 coreceptors in HCV entry [104, 105]. Next, a rate-limiting role for SR-BI in HCV infection has been reported [106]. The *in vitro* study demonstrated that increased SR-BI expression would enhance entry and internalization of HCV. Several studies investigating the postbinding cellular mechanisms found that the ability of HCV penetration into the hepatocytes may depend on clathrin-mediated endocytosis [107, 108]. After internalization, fusion occurs between viral and endosomal membranes. Recent assay further showed that the fusion may be low of pH and temperature dependent and facilitated by cholesterol [109, 110]. Although the detailed molecular mechanism of HCV entry still needs more investigations, it is conceivable that HCV internalization and fusion may offer many targets for the development of HCV entry inhibitors.

### 4.4. Promising Inhibitors against HCV Entry

Interfering with HCV entry into the hepatocytes holds great promises for the development of novel drugs. Several potential targets are suggested: (1) blocking virus-target cell interaction during attachment and binding; (2) interfering with postbinding events and (3) interfering with viral fusion. Various modalities may be adopted as HCV entry inhibitors: neutralizing antibodies for HCV envelope glycoproteins, blocking antibodies specific for host factors, and small molecular compounds against host factors or viral proteins.

CD81 is one of these potential targets. Imidazole-based compounds mimicking an alpha helix in the LEL of CD81 compete for HCV E2-CD81 binding [111]. These drugs bind E2 in a reversible manner and block E2-CD81 interaction while having no effect neither on CD81 expression nor on CD81 interaction with physiological partner molecules. In addition, recent study demonstrated that anti-CD81 antibodies completely protected human liver-uPA-SCID mice from a subsequent challenge with HCV consensus strains of different genotypes [112]. However, administration of anti-CD81 antibodies after viral challenge had no effect. Anti-SR-BI antibodies, which block the interaction of SR-BI with HCV, are another interesting strategy to prevent HCV entry. Anti-SR-BI antibodies have been demonstrated to inhibit HCVcc infection *in vitro* [113]. Moreover, small molecule inhibitors of SR-BI have recently been developed. For example, ITX5061 is a compound that inhibits entry of HCVpp from all major genotypes and HCVcc infection without affecting viral replication [114]. In addition, recent study demonstrated that a human monoclonal antibody targeting SR-BI efficiently precluded HCV infection and viral spread after LT *in vitro* and *in vivo* [115]. Kinetic studies suggest that this small molecule inhibitor targets HCV entry during an early postbinding stage. CLDN1 has been suggested to play an important role in cell-cell transmission. Recently, anti-CLDN1 antibodies inhibiting HCV infection *in vitro* have been developed [116]. The results have shown that anti-CLDN1 antibodies efficiently block cell entry of highly infectious escape variants of HCV that are resistant to host neutralizing antibodies. As mentioned above, EGFR and EphA2 are two newly established host factors for HCV entry. Thus, their respective inhibitors, erlotinib and dasatinib, are also regarded as promising HCV inhibitors in preventing HCV reinfection after LT [103].

In addition to cell surface expressed host factors, HCV internalization and fusion are complex processes that also offer several targets for antivirals. A recent study demonstrated that long phosphorothioate oligonucleotides (PS-ON) inhibited HCV internalization without affecting viral binding and replication [117]. The human liver-chimeric Alb-uPA/SCID mouse model also showed that PS-ON block *de novo* HCV infection. These data shed light on the promise of PS-ON as future clinical solution.

## 5. Conclusions

Recurrent HCV infection after liver transplantation has increasingly become a major clinical issue that the hepatologist and transplant surgeons should face. During the past decade, numerous therapeutic approaches based on both basic research and clinical evidences have been reported, but the effective strategy still needs more efforts to be approved. Meanwhile, many critical questions remain in several aspects of HCV, including the viral infection cycle activities, the detailed structure, composition, and function of the HCV virion in host cells. In addition, current standard of therapeutic formula consisting of Pegylated IFN combined with ribavirin and an NS3/4A inhibitor is ineffective or only partially effective. In fact, as reviewed in this paper, many novel inhibitors or neutralizing antibodies targeting HCV proteins and host factors hold promise to effectively prevent or treat HCV. Hopefully, these future strategies will improve the overall outcome of HCV recipients after liver transplantation [101].
